# Application of Integrated Optical Density in Evaluating Insulin Expression in the Endocrine Pancreas During Chronic Ethanol Exposure and β-Carotene Supplementation: A Novel Approach Utilizing Artificial Intelligence

**DOI:** 10.3390/ph17111478

**Published:** 2024-11-03

**Authors:** Cristian Sandoval, Luciano Canobbi, Álvaro Orrego, Camila Reyes, Felipe Venegas, Ángeles Vera, Francisco Torrens, Bélgica Vásquez, Karina Godoy, Mauricio Zamorano, José Caamaño, Jorge Farías

**Affiliations:** 1 Escuela de Tecnología Médica, Facultad de Salud, Universidad Santo Tomás, Los Carreras 753, Osorno 5310431, Chile; 2 Departamento de Ingeniería Química, Facultad de Ingeniería y Ciencias, Universidad de La Frontera, Temuco 4811230, Chile; mauricio.zamorano@ufrontera.cl; 3 Departamento de Medicina Interna, Facultad de Medicina, Universidad de La Frontera, Temuco 4811230, Chile; jose.caamano@ufrontera.cl; 4 Carrera de Tecnología Médica, Facultad de Medicina, Universidad de La Frontera, Temuco 4811230, Chile; l.canobbi01@ufromail.cl (L.C.); a.orrego03@ufromail.cl (Á.O.); c.reyes24@ufromail.cl (C.R.); f.venegas04@ufromail.cl (F.V.); a.vera08@ufromail.cl (Á.V.); 5 Institut Universitari de Ciència Molecular, Universitat de València, 46071 València, Spain; torrens@uv.es; 6 Departamento de Ciencias Básicas, Facultad de Medicina, Universidad de La Frontera, Temuco 4811230, Chile; belgica.vasquez@ufrontera.cl; 7 Centro de Excelencia en Estudios Morfológicos y Quirúrgicos (CEMyQ), Facultad de Medicina, Universidad de La Frontera, Temuco 4811230, Chile; 8 Núcleo Científico y Tecnológico en Biorecursos (BIOREN), Universidad de La Frontera, Temuco 4811230, Chile; karina.godoy@ufrontera.cl

**Keywords:** alcohol intake, insulin, antioxidant treatment, chronic alcohol consumption

## Abstract

Background: β-carotene is an essential antioxidant, providing protection against type 2 diabetes mellitus, cardiovascular illnesses, obesity, and metabolic syndrome. This study investigates the impact of β-carotene on biochemical parameters and pancreatic insulin expression in mice exposed to ethanol. Methods: Thirty-six C57BL/6 mice (*Mus musculus*) were divided into six groups: 1. C (control), 2. LA (3% alcohol dose), 3. MA (7% alcohol dose), 4. B (0.52 mg/kg body weight/day β-carotene), 5. LA+B (3% alcohol dose + 0.52 mg/kg body weight/day β-carotene), and 6. MA+B (7% alcohol dose plus 0.52 mg/kg body weight/day β-carotene). After 28 days, the animals were euthanized for serum and pancreatic tissue collection. Biochemical analysis and pancreatic insulin expression were performed. One-way ANOVA was used. Results: The B, LA+B, and MA+B groups improved insulin levels and decreased HOMA-β versus the C group, with the LA+B and MA+B groups also showing lower ADH and ALDH levels than their nonsupplemented counterparts (*p* < 0.05). The B, LA+B, and MA+B groups showed a greater β-cell mass area compared to the unsupplemented groups. Additionally, the LA+B and MA+B groups demonstrated significantly increased β-cell area and integrated optical density compared to the LA and MA groups, respectively (*p* < 0.001). Conclusions: In mice, β-cell loss led to increased glucose release due to decreased insulin levels. β-carotene appeared to mitigate ethanol’s impact on these cells, resulting in reduced insulin degradation when integrated optical density was used. These findings suggest that antioxidant supplementation may be beneficial in treating ethanol-induced type 2 diabetes in animal models.

## 1. Introduction

Alcohol consumption poses a significant public health concern, with an estimated annual cost of EUR 155 billion attributed to related issues [1]. It is widely recognized as a major risk factor for both acute and chronic pancreatitis, contributing to approximately 50% to 80% of all documented cases [2]. Recent research highlights alcohol as the predominant factor responsible for pancreatitis in the United States [3].

However, studies indicate that less than 5% of individuals who chronically consume large amounts of alcohol are at risk of developing pancreatitis [4]. This discrepancy suggests that the development of alcohol-induced pancreatitis may require additional risk factors, influenced by genetic or environmental variables [5,6].

The complex and multifaceted effects of alcohol on the pancreas contribute to an incomplete understanding of the etiology of alcohol-induced pancreatitis. Alcohol is believed to damage pancreatic acinar cells, ductal epithelium, and stellate cells, potentially promoting pancreatic fibrosis [7,8]. Alcohol-induced pancreatitis is likely to develop only when compensatory mechanisms are exhausted or when other genetic or environmental stresses increase pancreatic vulnerability. Findings from animal models support this hypothesis, suggesting that the pancreas can mitigate alcohol-induced damage by initiating an adaptive stress response [9,10].

Pancreatic acinar cells metabolize alcohol through both oxidative and nonoxidative pathways [11,12,13,14,15]. The metabolic breakdown of alcohol in these cells generates toxic byproducts, including fatty acid ethyl esters (FAEEs), acetaldehyde, and reactive oxygen species (ROS), which contribute to cellular damage [11,16,17]. This oxidative stress destabilizes zymogen granules and lysosomes, as well as disrupts other organelle functions within the cell [4,11,18].

Chronic pancreatitis is marked by recurring abdominal pain and is frequently accompanied by symptoms such as nausea and weight loss [19]. Persistent pancreatic damage reduces the secretion of enzymes essential for digestion and fat absorption, leading to a progressive decline in digestive function. Additionally, the destruction of pancreatic β-cells, which produce, store, and release insulin, increases the risk of diabetes development [20].

Diabetes mellitus (DM) is a common chronic metabolic disorder characterized by elevated blood glucose levels, arising from either insufficient insulin production or impairments in insulin signaling [21,22]. As a growing global public health challenge, diabetes incidence is anticipated to rise significantly in the coming decades, driven in part by aging populations worldwide [23].

Both type 1 and type 2 diabetes (T2DM) involve pancreatic dysfunction, impairing the β-cells’ capacity to meet increased insulin demands [24]. A family history of diabetes is widely recognized as a significant risk factor for developing T2DM and pre-diabetes [25,26,27].

Evidence suggests that β-carotene supplementation enhances GSH concentration by stimulating the activity of GSH synthetase. Previous research has shown that adding β-carotene can stop the loss of GSH caused by ethanol by raising the amount of GSH inside cells [28,29]. These studies indicate that it may be due to the stimulation of GSH synthetase activity caused by β-carotene. Therefore, β-carotene supplementation may serve as an effective approach to mitigate liver damage resulting from excessive alcohol consumption and to prevent the progression of alcoholic disease to more severe conditions [14].

Previous studies have shown that ethanol exposure induces oxidative stress and apoptosis, leading to tissue damage, while β-carotene supplementation (0.52 mg/kg BW/day) can alleviate ethanol-induced injury by reducing oxidative stress and inhibiting apoptosis in tissues [30]. Additional findings also report an increase in serum total amylase levels and a decrease in lipase levels in groups receiving 7% alcohol plus β-carotene supplementation compared to those without it [15]. Histological analysis further revealed that perilobular parenchyma, intralobular parenchyma, and fibrosis scores were lower in the 7% alcohol plus β-carotene group compared to the groups given 3% alcohol, 7% alcohol, or 3% alcohol plus β-carotene [15]. These findings suggest that antioxidant therapy could be beneficial in addressing the effects of ethanol exposure in animal models.

β-carotene, a member of the carotene family, is among the most prevalent carotenoids in food sources and is also present in the human body. About 17–45% of the consumed β-carotene remains intact in organisms, indicating its significant bioavailability, which refers to its effective absorption and utilization capacity [31]. Research indicates that β-carotene serves as a safeguard against several conditions, including type 2 diabetes mellitus (T2DM), cardiovascular disease, obesity, and metabolic syndrome (MetS) [32,33,34]. β-carotene is noted for its ability to enhance expression and secretion, which in turn improves insulin sensitivity [35].

The consumption of β-carotene is linked to several outcomes, including a decrease in the size of adipocytes and overall body adipose tissue; a decline in proinflammatory markers, low-density lipoprotein cholesterol (LDL-c), and very-low-density lipoprotein cholesterol (VLDL-c); and an increase in high-density lipoprotein cholesterol (HDL-c) [36,37,38,39]. Furthermore, they have the potential to enhance insulin resistance and maintain insulin receptors [37,39]. These actions take place to manage oxidative stress, which plays a role in all of the diseases mentioned [40].

β-carotene has the ability to modulate lipid and carbohydrate metabolism, thereby enhancing the function of pancreatic cells and improving hyperglycemic conditions. The regulation of β-pancreatic cell functions stimulates insulin secretion, regulates lipid metabolism, and alleviates oxidative and inflammatory stress [39,41,42].

The American Diabetes Association defines diabetes as a condition marked by the degeneration of insulin- and glucagon-producing cells. Histological examination reveals fibrosis, the shrinkage of pancreatic acini, chronic inflammation, the deformation of pancreatic ducts with narrowed regions, and damage to ß- and α-cells [14,15,43].

Thus, a comprehensive histological evaluation is essential. In particular, assessing insulin activity requires a multifaceted approach involving the analysis of serum biochemical markers alongside the measurement of insulin production in pancreatic islets [15]. This assessment may include evaluating the dimensions of the pancreatic islets or calculating their integrated optical density (IOD), with average values typically presented per cell [44,45]. The final evaluation involves staining the pancreatic islets with specific markers, such as immunohistochemistry staining, followed by image capture with a light microscope equipped with specialized optical filters. The IOD quantifies the light absorption across the entire pancreatic islet as captured in the images.

The cellular IOD serves as a quantitative metric. Previous research utilizing immunohistochemistry and Sirius Red staining on liver tissue observed significant increases in IOD levels following moderate alcohol consumption and carotene supplementation [14]. Additionally, IOD can be instrumental in identifying morphologically and functionally distinct cell subpopulations by maintaining consistent light contrast in imaging [14,45]. Consequently, the IOD analysis of cell dynamics may correlate with various pathophysiological states, offering valuable insights into disease processes.

Previous research has documented the effects of alcohol on the pancreas; however, the potential benefits of antioxidant supplementation during chronic ethanol exposure remain insufficiently understood. This study aimed to evaluate glucose and insulin serum levels, as well as insulin expression within the endocrine pancreas, in C57BL/6 mice subjected to ethanol exposure and/or β-carotene supplementation.

## 2. Results

### 2.1. Biochemistry

Table 1 presents the biochemical analyses of insulin, glucose, ADH, ALDH, HOMA-β, and HOMA-IR indices. The LA+B group showed significantly lower HOMA-β levels than the C, LA, MA, and B groups, while the MA+B group displayed reduced insulin and HOMA-β levels but higher glucose levels relative to the C group (*p* < 0.05). β-carotene supplementation improved the insulin levels and decreased HOMA-β versus the C group, with the LA+B and MA+B groups also showing lower ADH and ALDH levels than their nonsupplemented counterparts (*p* < 0.05).

### 2.2. Immunohistochemistry

**Figure 1:**
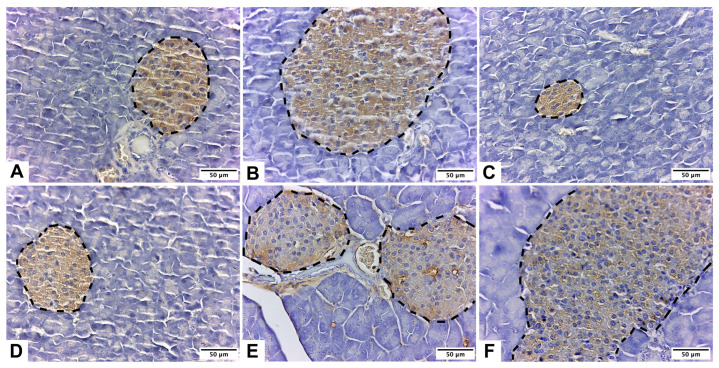
Pancreas of male C57BL/6 mice stained with anti-insulin IgG Guinea pig primary antibody. Insulin expression in the endocrine pancreas was observed in groups: control (**A**); low-dose alcohol (**B**); moderate-dose alcohol (**C**); β-carotene (**D**); low-dose alcohol + β-carotene (**E**); and moderate-dose alcohol + β-carotene (**F**). Pancreatic islets are in the segmented lines.

The immunohistochemical localization of insulin was observed in the endocrine pancreas across all the experimental groups (Figure 1). The histological analysis revealed variations in immunolabeling patterns and intensity among the groups. The C group exhibited a normal morphology of the beta-pancreatic islets, with the cells showing strong positive immunostaining (Figure 1A).

Figure 1D–F illustrate an association between β-carotene supplementation and an increase in both the number and size of the pancreatic islets. Notably, no insulin marker immunoreactivity was detected outside the beta-pancreatic islets or the endocrine pancreas. Among the groups, the MA+B group displayed the highest immunostaining intensity (Figure 1D).

### 2.3. Area

The groups supplemented with β-carotene (B, LA+B, and MA+B) exhibited at least a 30% increase in the β-cell mass area compared to those without β-carotene supplementation (C, LA, and MA) (Table 2). In ethanol-consuming mice (LA and MA vs. LA+B and MA+B, respectively), β-carotene supplementation led to a 30–50% restoration of the median β-cell mass relative to the C and MA+B groups (*p* < 0.05).

### 2.4. Integrated Optical Density Analysis

The immunohistochemistry analysis demonstrated the immunolabeling of β-cells throughout different regions of the beta-pancreatic islets in all the experimental groups. The concentration and intensity of insulin within the islets were higher in the groups supplemented with β-carotene (Table 2).

## 3. Discussion

### 3.1. Summary of Key Findings and Interpretation

Studies have shown a positive correlation between higher alcohol consumption and an increased incidence of T2DM [46]. In contrast, research also suggests that moderate alcohol intake may be linked to a reduced risk of T2DM [47]. Animal model studies investigating insulin production after ethanol exposure indicate that antioxidants can be effective in managing elevated blood glucose levels [14,48].

Although the specific mechanisms through which alcohol influences insulin remain unclear, numerous studies have identified a U-shaped or J-shaped relationship between alcohol consumption and insulin sensitivity or plasma insulin concentrations [49,50,51,52]. In individuals with T2DM, insulin production continues during the early stages of the disease; however, the body becomes resistant to insulin’s effects, as reflected in our results. Initially, the pancreas compensates for this resistance by increasing insulin synthesis, but eventually, it reaches a threshold where it can no longer produce adequate insulin.

### 3.2. Biochemistry

The HOMA-IR is considered a straightforward, cost-effective, and trustworthy indicator of insulin resistance, whereas the HOMA-β index has been identified as an effective measure of β-cell function [53]. Our results show that the groups that regularly consumed low and moderate amounts of ethanol had a lower HOMA-IR index and better insulin sensitivity (Table 1), which is in line with what other researchers have found. Nonetheless, the HOMA-β index in the mice subjected to prolonged ethanol consumption varies from what has been previously stated.

Chronic alcoholics experience an increase in alcohol metabolism [54]. This is often regarded as a significant factor contributing to alcohol-induced injury [55]. The primary enzymes involved in alcohol metabolism include alcohol dehydrogenase (ADH), mitochondrial aldehyde dehydrogenase (ALDH), and cytochrome P450 2E1 (CYP2E1). The alcohol is broken down into acetaldehyde by ADH and then further converted into acetate by ALDH. Therefore, significant toxicity caused by ethanol may be linked to the functions of ADH and ALDH [56].

### 3.3. Immunohistochemistry

Immunocytochemistry is a highly effective and sensitive method for detecting insulin and assessing its expression levels in pancreatic islets [57]. In this study, immunohistochemical labeling proved to be a valuable tool for identifying and distinguishing insulin-expressing cells in the pancreatic islets of C57BL/6 mice. The results showed that the islets predominantly contain insulin-producing cells with a lobular shape, consistent with the findings from previous studies [58,59].

This study employed ethanol exposure to induce diabetes in C57BL/6 mice. In the male mice of this strain, ethanol administration leads to elevated blood glucose levels, indicating that ethanol exerts a cytotoxic effect on pancreatic β-cells, likely through the generation of free radicals. The resulting destruction of β-cells reduces or entirely depletes insulin, thereby causing hyperglycemia [60]. However, in the mice that received β-carotene supplementation alongside ethanol, there was a notable increase in both the number and size of pancreatic islets (Figure 1D–F). The MA+B group exhibited the highest immunostaining intensity among the groups, as shown in Figure 1D. These findings suggest that β-carotene, as an antioxidant, may reduce free radical presence and mitigate ethanol’s cytotoxic effects on pancreatic cells.

### 3.4. Integrated Optical Density Analysis

Analyzing the mean gray value of each object within a digital image allows for the calculation of optical density (OD), a measurable parameter. Integrated optical density (IOD) represents the relationship between OD and a specific area of an image [61]. Pixel density quantifies the number of pixels per unit area, enabling an assessment of the object of interest relative to the image background. IOD facilitates the evaluation of immunohistochemical labeling by measuring color variations at the pixel level, which are then converted into numerical values to establish a quantifiable parameter.

In C57BL/6 mice, Table 2 presents the measured area and IOD characteristics of pancreatic islets. IOD properties varied across the experimental groups, with each group demonstrating statistically significant differences from the C group (*p* < 0.05). Notably, the β-cell mass area was larger in the β-carotene-supplemented groups (B, LA+B, and MA+B) compared to the unsupplemented groups (C, LA, and MA) (Table 2). Additionally, the β-carotene-supplemented groups showed elevated insulin concentration and potency in the islets, as displayed in Table 1.

Insulin resistance is defined as an inadequate response of tissues to insulin’s action in the bloodstream and is widely recognized as a key indicator for the development of metabolic disorders such as T2DM and metabolic syndrome [62]. Previous studies have explored the relationship between carotenoid consumption and impaired glucose tolerance, finding an inverse linear correlation between the concentrations of β-carotene and lycopene and the level of glucose tolerance, as shown by glucose tolerance test results [59]. These findings suggest that carotenoids may improve insulin sensitivity. Studies by Facchini et al. and Sugiura et al. have shown that lower plasma carotenoid levels are associated with higher insulin resistance in healthy individuals [63,64].

In this study, insulin expression in the pancreatic islets varied among the groups. The LA group demonstrated increased β-cell area and IOD (270.44 ± 107.99 μm^2^ and 35661.82 ± 13921.15 lum/μm^2^, respectively) compared to the C group (194.78 ± 38.30 μm^2^ and 26006.67 ± 5063.97 lum/μm^2^, respectively), potentially indicating heightened insulin sensitivity (*p* < 0.001). Although no significant differences in the serum glucose levels were found between the LA and C groups using the same experimental models, the LA group exhibited lower serum insulin content relative to the C group [60].

When β-carotene was administered concurrently with ethanol (in the LA+B and MA+B groups), there was a significant increase in the β-cell area and IOD compared to the groups without the β-carotene supplementation (LA and MA), although no differences were observed in the serum insulin or glucose levels. This effect could be attributed to several potential mechanisms: (1) β-carotene may reduce ethanol-induced β-cell damage while increasing insulin sensitivity [11,12,16]; (2) β-carotene alone may enhance insulin sensitivity [52,65,66]; or (3) β-carotene could mitigate ethanol damage in β-cells while jointly enhancing insulin sensitivity with ethanol [11,12,16,52,65,66].

### 3.5. Limitations

Our data offer new insights into the relationship between alcohol-induced diabetes and antioxidant therapies, specifically β-carotene. However, this study has a limitation in that it does not examine the interactions between alcohol metabolism byproducts and other pancreatic hormones, such as glucagon, somatostatin, amylin, or pancreatic polypeptide, in the context of antioxidant treatments. Future research should, therefore, focus on investigating the effects of β-carotene in cultured pancreatic cells and identifying the specific molecules and signaling pathways involved. Nevertheless, our findings suggest that β-carotene exposure may mitigate ethanol-induced damage to β-cells and enhance insulin sensitivity, even during ethanol consumption.

## 4. Materials and Methods

### 4.1. Sample Size

The study was a comparative analysis of independent groups, with parameters set at an alpha of 0.10, beta of 0.05, standard deviation of 0.05, a minimum detectable difference between groups of 0.1, and an anticipated follow-up loss proportion of 0.2 [67,68,69]. The sample size calculation was conducted using the G*Power 3.1.9.7 Software (Heinrich-Heine-Universität Düsseldorf, Düsseldorf, Germany).

### 4.2. Animals

Thirty-six male C57BL/6 mice (*Mus musculus*), aged fifty days, were obtained from the Public Health Institute of Chile. They were housed in the Animal Facility at the Center of Excellence in Morphological and Surgical Studies (CEMyQ), Universidad de La Frontera, for 30 days to acclimate to their environment. During this period, the mice were provided with a standard laboratory diet (AIN-93M) and water ad libitum. Lighting conditions were maintained on a 12 h light/dark cycle from 08:00 to 20:00 and 20:00 to 08:00. Animal care followed the guidelines from the Institute for Laboratory Animal Research’s Committee for the Update of the Guide for the Care and Use of Laboratory Animals [70].

**Figure 2:**
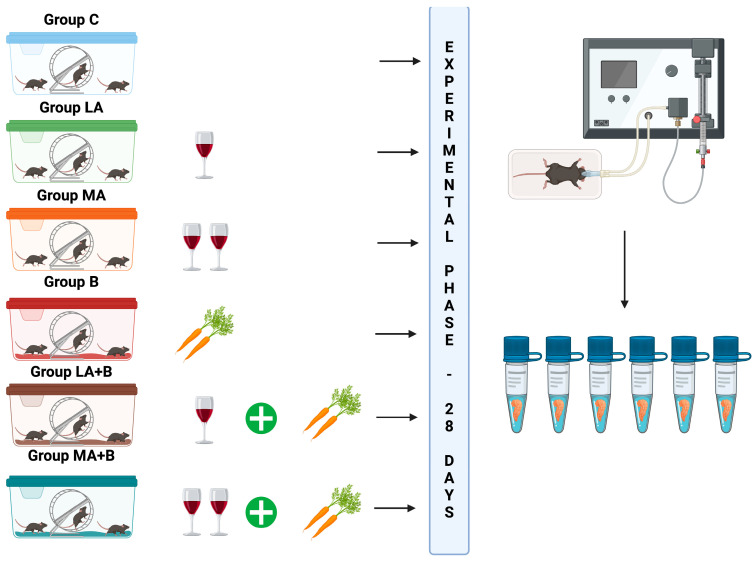
Summary of the experimental design of the study. In this schematic representation, one glass of wine corresponds to an administration of a 28-day 3% alcohol dose, and two glasses of wine correspond to an administration of a 28-day 7% alcohol dose. Also, the carrots correspond to an administration of 28-day 0.52 mg/kg body weight/day of β-carotene. After the experimental phase, the animals were euthanized and samples (the blood and tissues) were obtained.

On the first day of the experiment, the mice were divided into six groups (n = 6 per group):

**Control group (Group C):** No alcohol or β-carotene administration.
**Low-dose alcohol group (Group LA):** Administered 3% *v*/*v* alcohol ad libitum for 28 days based on studies suggesting low alcohol intake enhances insulin sensitivity [49,71,72,73].
**Moderate-dose alcohol group (Group MA):** Administered 7% *v*/*v* alcohol ad libitum for 28 days, as moderate intake is also linked to increased insulin sensitivity [49,71,72,73].
**β-carotene group (Group B):** Administered 0.52 mg/kg body weight/day β-carotene for 28 days, as this dose has been shown to mitigate alcohol-induced liver damage by reducing oxidative stress and inhibiting apoptosis [30].
**Low-dose alcohol + β-carotene group (Group LA+B):** Administered low-dose alcohol plus 0.52 mg/kg body weight/day β-carotene for 28 days.
**Moderate-dose alcohol + β-carotene group (Group MA+B):** Administered moderate-dose alcohol plus 0.52 mg/kg body weight/day β-carotene for 28 days. Figure 2 illustrates the experimental design.

Chronic ethanol was administered using a modified Lieber–DeCarli liquid diet [71,73], while β-carotene was provided orally at a dosage of 0.52 mg/kg body weight per day [15]. This experimental model has been previously applied in animal studies [14,15,18,60].

### 4.3. Alcoholism and Treatments

A chronic plus single binge ethanol consumption (referred to as Lieber–DeCarli alcoholic diet) was used [71,74,75].

The ethanol groups were provided a liquid diet containing either 3% (LA or LA+B) or 7% (MA or MA+B) alcohol over a period of 28 days, while the control groups (C or B) were pair-fed an equivalent control diet for the same duration. On day 29, the mice in the ethanol groups were administered a single oral dose of ethanol (5 g/kg body weight, 20% ethanol), while the control groups received isocaloric dextrin maltose. Ethanol was sourced from Merck KGaA (107017, Darmstadt, Germany). The liquid diets were freshly prepared and administered daily [76].

β-carotene was administered in a dose of 0.52 mg/kg body weight/day (C9750, Sigma-Aldrich Co., St. Louis, MO, USA). β-carotene was diluted in water for the groups C, LA, MA, and B, whereas alcohol was used for the dissolution in the groups LA+B and MA+B. β-carotene was administered once a day by oral gavage (C9750, Sigma-Aldrich Co., St. Louis, MO, USA).

### 4.4. Euthanasia

On day 28, the animals were deprived of food for 6 h and then euthanized with sodium pentobarbital.

### 4.5. Biochemical Analyses

Centrifugation at 3500 rpm for 15 min separated the serum, which we then stored at −80 °C until analysis. In the biochemical analysis, we used a colorimetric kit (Sigma-Aldrich Co., St. Louis, MO, USA) to quantify the physiological concentration of glucose and a mouse-specific ELISA kit (Sigma-Aldrich Co., St. Louis, MO, USA) to measure insulin levels. The enzymatic activity of ADH and ALDH was assessed using the respective kit provided by Sigma-Aldrich Co. (St. Louis, MO, USA).

### 4.6. Homeostasis Model Assessment of β-Cell Function (HOMA-SS)

The HOMA-ß index was determined using Equation (1) [77]:

(1) $$\frac{\left[20\times \mathrm{fasting}\;\mathrm{insulin}\;\left(\frac{\mathrm{uU}}{\mathrm{mL}}\right)\right]}{\left[\mathrm{fasting}\;\mathrm{glucose}\left(\frac{\mathrm{mmoL}}{L}\right)-3.5\right]}$$

### 4.7. Homeostasis Model Assessment of Insulin Resistance (HOMA-IR)

The HOMA-IR index was determined using Equation (2) [78]:

(2) $$\frac{\left[\mathrm{fasting}\;\mathrm{insulin}\left(\frac{\mathrm{uU}}{\mathrm{mL}}\right)\times \mathrm{fasting}\;\mathrm{glucose}\;\left(\frac{\mathrm{mmoL}}{L}\right)\right]}{22.5}$$

### 4.8. Processing and Staining of Pancreas

To ensure random sampling, multiple sections were obtained from each pancreas, capitalizing on the tissue’s isotropic characteristics. After a 48 h fixation in 4% buffered formalin (1.27 mol/L formaldehyde in 0.1 M phosphate buffer, pH 7.2; Sigma-Aldrich, St. Louis, MO, USA), the samples were dehydrated and embedded in Paraplast Plus (Sigma-Aldrich, St. Louis, MO, USA). Each block was sectioned into four groups, with 5 μm cuts spaced 120 μm apart using a microtome (Leica^®^ RM2255, Leica Biosystems, Nussloch, Germany).

### 4.9. Immunohistochemistry

**Figure 3:**
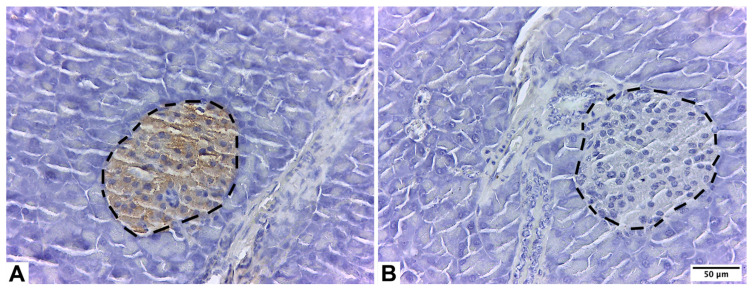
Exocrine pancreas of male C57BL/6 mice. Positive control for pancreatic islets stained with anti-insulin IgG Guinea pig primary antibody (**A**); negative control for pancreatic islets stained with primary antibody (anti-insulin IgG Guinea pig primary antibody was omitted) (**B**). Pancreatic islets are in segmented lines.

In summary, the paraffin sections were hydrated using a series of alcohols in decreasing concentrations following the standard procedure for traditional histological staining. Subsequently, they were immersed in distilled water for a duration of five minutes to restore their moisture content. Each histological section was washed in 1× PBS (Sigma-Aldrich Co., St. Louis, MO, USA) twice. They were treated with H_2_O_2_ (*v*/*v*) (ab64264, Abcam, Cambridge, UK) for 15 min to block the activity of endogenous peroxidase. Then, antigenic recovery was performed with HistoReveal (ab103720, Abcam, Cambridge, UK) for 10 min at room temperature. Next, each histological section was washed in 1× PBS three times. Then, the unspecific background was blocked using Protein Block (ab64207, Abcam, Cambridge, UK) for 15 min at room temperature. Each washing was performed with 1× PBS three times. First, the sections were incubated with anti-insulin IgG Guinea pig primary antibody (ab7842, Abcam, Cambridge, UK), dilution 1:50, in PBS overnight at 4 °C under a wet chamber. Second, each washing was performed with 1× PBS, four times. After washing with PBS, the sections were incubated with a biotinylated goat anti-polyvalent antibody (ab64207, Abcam, Cambridge, UK) for 10 min at room temperature in a wet chamber. Next, each wash was performed with 1× PBS four times. Then, the sections were incubated with streptavidin peroxidase (ab64207, Abcam, Cambridge, UK) for 10 min at room temperature. Afterward, each washing was performed with 1× PBS, four times. Finally, they were incubated with diaminobenzidine-peroxidase (ab64207, Abcam, Cambridge, UK) for visualization for ten minutes and washed with 1× PBS four times. The nuclear counterstain was performed with Harris hematoxylin (Sigma-Aldrich Co., St. Louis, MO, USA) for 50 s. The slides were dehydrated using a series of alcohols (Sigma-Aldrich Co., St. Louis, MO, USA) in increasing concentrations, following the standard procedure for traditional histological staining. A total of four pancreatic islets by slide were observed under a light microscope (Leica^®^ LED750, Leica Biosystems, Nussloch, Germany) and photographed (Leica^®^ ICC50W, Leica Biosystems, Nussloch, Germany). A total of 144 pancreatic islets were evaluated. For each immunohistochemical reaction, negative controls were used, which were incubated in PBS, omitting the primary antibody (ab7842, Abcam, Cambridge, UK). Table 3 shows the labeled process used for positive and negative control. The immunolabeling was quantified by the area (mm^2^) occupied in each field, and the IOD analysis was expressed as lum/μm^2^ [79]. Both measurements were made using the Image-ProPremier 9.1 software (Media Cybernetics, Warrendale, PA, USA). Figure 3 shows the positive and negative controls.

### 4.10. Statistical Analysis

Levene’s test was applied to assess the homoscedasticity of variances, while the Kolmogorov–Smirnov test was used to evaluate data normality. These tests were conducted to assess disparities in the quantitative data. Group differences were analyzed using one-way ANOVA, followed by either Dunnett’s T3 test or Tukey’s post hoc HSD test, as appropriate. Statistical significance was determined at a *p*-value of less than 0.05 using IBM SPSS Statistics, Version 21 (IBM Corp., Armonk, NY, USA).

## 5. Conclusions

Insulin expression in pancreatic islets varied among the groups. In mice, β-cell loss led to increased glucose release due to decreased insulin levels. β-carotene appeared to mitigate ethanol’s impact on these cells, resulting in reduced insulin degradation. In the pancreatic islets of the C57BL/6 mice, β-carotene exposure improved insulin sensitivity and lessened ethanol-induced β-cell damage. These findings suggest that antioxidant supplementation may be beneficial in treating ethanol-induced T2DM in animal models. Further research is necessary to better understand the interaction between alcohol consumption and antioxidant therapy, including studies using specific cell lines and clinical trials.

## Figures and Tables

**Table 1:** Biochemical evaluation of male C57BL/6 mice following ethanol consumption and β-carotene supplementation.

Media ± SD							
	C (n = 6)	LA (n = 6)	MA (n = 6)	B (n = 6)	LA+B (n = 6)	MA+B (n = 6)	*p*
Insulin (μU/mL)	28.60 ± 5.45	16.44 ± 7.27 ^a^	14.96 ± 5.01 ^a^	24.27 ± 9.58 ^abc^	11.34 ± 3.91 ^ad^	9.46 ± 1.55 ^acd^	<0.001
Glucose (mmol/L)	9.03 ± 0.53	13.45 ± 1.27 ^a^	15.11 ± 0.45 ^ab^	14.63 ± 0.84 ^ab^	13.46 ± 0.43 ^acd^	14.31 ± 0.86 ^a^	<0.001
ADH (nmol/min/mL)	20.42 ± 2.52	33.03 ± 3.42 ^a^	36.15 ± 2.74 ^a^	23.22 ± 5.58 ^bc^	26.09 ± 3.16 ^abc^	29.83 ± 3.51 ^acd^	<0.001
ALDH (pmol/min/mL)	11.81 ± 1.82	15.81 ± 1.62 ^a^	17.04 ± 1.78 ^a^	11.21 ± 1.30 ^bc^	14.25 ± 1.67 ^acd^	15.81 ± 1.77 ^ad^	<0.001
HOMA-β	104.41 ± 21.30	33.34 ± 15.51 ^a^	25.77 ± 6.77 ^a^	43.53 ± 15.51 ^a^	22.77 ± 7.38 ^a^	17.61 ± 3.19 ^ad^	<0.001
HOMA-IR	11.44 ± 1.49	9.83 ± 4.59	10.06 ± 2.76	15.84 ± 6.04	6.79 ± 2.21 ^d^	6.01 ± 0.85 ^d^	<0.001

^a^ significant differences (*p* < 0.05) with the C group. ^b^ significant differences (*p* < 0.05) with the LA group. ^c^ significant differences (*p* < 0.05) with the MA group. ^d^ significant differences (*p* < 0.05) with the B group. Differences were analyzed by one-way ANOVA.

**Table 2:** Analysis of area and integrated optical density properties of β-cell in pancreatic islets of C57BL/6 mice.

Media ± SD							
	C (n = 6)	LA (n = 6)	MA (n = 6)	B (n = 6)	LA+B (n = 6)	MA+B (n = 6)	*p*
Area (μm^2^)	194.78 ± 38.30	270.44 ± 107.99 ^a^	69.24 ± 22.61 ^ab^	429.50 ± 246.77 ^ac^	330.79 ± 117.64 ^ac^	1028.27 ± 356.19 ^ac^	<0.001
Integrated optical density (lum/μm^2^)	26,006.67 ± 5063.97	35,661.82 ± 13,921.15 ^a^	9722.07 ± 3049.34 ^ab^	51,611.28 ± 29,343.24 ^ac^	43,537.44 ± 15,480.09 ^ac^	54,324.13 ± 29,600.55 ^ac^	<0.001

^a^ significant differences (*p* < 0.05) with the C group. ^b^ significant differences (*p* < 0.05) with the LA group. ^c^ significant differences (*p* < 0.05) with the MA group. A total of 144 pancreatic islets were evaluated, 24 pancreatic islets by group. Differences were analyzed by one-way ANOVA.

**Table 3:** Labeled process used for positive and negative controls to determine insulin in pancreatic islets of C57BL/6 mice.

	Block Peroxidase	Antigenic Recovery	Unspecific Background	Primary Antibody	Secondary Antibody	Labeled	Detection
Positive control (protein of interest)	H_2_O_2_	HistoReveal	Protein Block	Anti-insulin IgG guinea pig primary antibody	Biotinylated goat anti-polyvalent antibody	Streptavidin peroxidase	Diaminobenzidine-peroxidase
Negative control (without protein of interest)	H_2_O_2_	HistoReveal	Protein Block	Saline solution	Biotinylated goat anti-polyvalent antibody	Streptavidin peroxidase	Diaminobenzidine-peroxidase
Samples (pancreas cuts)	H_2_O_2_	HistoReveal	Protein Block	Anti-insulin IgG guinea pig primary antibody	Biotinylated goat anti-polyvalent antibody	Streptavidin peroxidase	Diaminobenzidine-peroxidase

## Data Availability

The original data presented in the study are openly available in FigShare at https://doi.org/10.6084/m9.figshare.27130587.v1 (accessed on 2 November 2024).
